# Molecular Dietary Analysis of Three Sympatric Mustelidae in Northeast China

**DOI:** 10.3390/ani12233290

**Published:** 2022-11-25

**Authors:** Dong Zhao, Zhihui Liu, Mengyu Tan, Yue Wang, Wenqian Dai, Jianping Ge, Limin Feng

**Affiliations:** Ministry of Education Key Laboratory for Biodiversity Science and Engineering, National Forestry and Grass-Land Administration Key Laboratory for Conservation Ecology of Northeast Tiger and Leopard National Park, Northeast Tiger and Leopard Biodiversity National Observation and Research Station, National Forestry and Grassland Administration Amur Tiger and Amur Leopard Monitoring and Research Center, College of Life Sciences, Beijing Normal University, Beijing 100875, China

**Keywords:** dietary analysis, sympatric Mustelidae, fecal DNA, species conservation

## Abstract

**Simple Summary:**

Mesopredators (i.e., carnivores of small or intermediate body size) are key species for ecosystem stability, especially when larger carnivore populations have declined, as they have important ecosystem functions, such as population size control. They are essential for ecosystem energy circulation. Studies of predators’ dietary habits and dietary differentiation between species provide critical information for identifying the structure and dynamics of trophic interactions in an ecosystem. Past research studies focused only on the diet of large carnivores while few detailed dietary datasets of mesopredators exist that present knowledge gaps regarding their coexistence. Using DNA metabarcoding, we studied the prey of three sympatric Mustelidae species in the Northeast Tiger and Leopard National Park. We find that prey preferences were largely partitioned by the three Mustelidae species and that they likely competed over certain dietary items (e.g., frogs and rodents). Our results provide an initial characterization of prey relationships between different Mustelidae species and may be used to inform management on dietary preferences of mesopredators that are affected by anthropogenic activities. Although human disturbance has declined with the creation of the national park, extra management for the protection of ecosystem functions and food webs is still needed.

**Abstract:**

Diet analysis is essential to fully understand the biology of a species and its function within the ecosystem, as well as being key in identifying food web interactions and the population dynamics of predators and prey. The understanding of the diet of small to mid-sized carnivores remains generally lacking or uninformative due to the inability for taxonomic resolution based on morphology. The yellow-throated marten (*Martes flavigula*), Eurasian river otter (*Lutra lutra*), and Siberian weasel (*Mustela sibirica*) are three important Mustelidae species in ecosystems of northeast China. Based on fecal DNA and a next-generation sequencing (NGS) approach, we analyzed the vertebrate prey of these three sympatric Mustelidae. Prey included 7 mammalian taxa, 10 fishes, 2 birds, and 2 amphibians, with 85% of the taxa assigned to the species level. In total, twenty-one vertebrate prey taxa were identified from seven yellow-throated martens, eight Eurasian river otters, and two Siberian weasels. Concerning identified dietary species, 10 taxa were consumed by yellow-throated martens, 14 by Eurasian river otters, and 4 by Siberian weasels. Some prey species were identified in more than one species. Amphibians and fishes were the most dominant Eurasian river otter prey categories, whereas Eurasian badger (*Meles leucurus*), birds, and rodents were the main yellow-throated marten prey; amphibians and rodents were largely contained in Siberian weasel prey. Among prey items, Dybowski’s frog (*Rana dybowskii*) and Korean field mouse (*Apodemus peninsulae*) were identified in all three Mustelidae species but our analyses suggest potential diet preferences among Mustelidae species. Future studies should focus on understanding the trophic relationships of these three Mustelidae species, providing valuable information for their conservation planning.

## 1. Introduction

Understanding predator–prey interactions is one of the major goals in ecological studies [1]. Studying the details of a species’ diet not only sheds light into their status within the food web but also provides a baseline for comparative purposes as environments continue to change [2]. Knowledge of a species’ diet can provide valuable information for developing specific conservation plans and management strategies [3]. In many situations prey choice by predators in the field cannot be established or quantified using direct observation. The remains of some prey may be visually identified in the guts and feces of predators but not all predators ingest such hard remains and even those that do consume them may also ingest soft-bodied prey that leave no recognizable remnants [2]. DNA-based methods are powerful tools with the potential to avoid these problems in studying food chains. They can also be used to identify prey when the remains in the fecal samples are degraded or lack solid parts [2].

Large mammalian carnivores (adult body mass ≥ 15 kg) typically function as apex predators in food webs [4] and are key regulators of community structure through top-down control of trophic cascades [5]. Small to mid-sized carnivores that often occupy the middle-rank in the predator hierarchy (i.e., mesopredators) tend to have more diverse and flexible diets, compared to those of large carnivores [6]. Hence, their coexistence mechanisms, especially between multiple sympatric Mustelidae species, are important for ecosystem stability. Mustelidae is one of the largest family of the eight carnivore families [7], distributed throughout Oceania. They live in diverse habitats, including terrestrial and aquatic habitats [8], which suggests they can affect the ecosystem through cascading effects. Here, we focused on the study of three sympatric Mustelidae species in China.

The yellow-throated marten (*Martes flavigula*) is one of the living species of the genus *Martes*, which is distributed throughout central and southern Asia in a wide variety of habitats [9]. Despite its extensive geographical range in China, the diet of this species has so far received little attention, only reported in the Sichuan province in the southwest of China [10]. This previous study showed that the content of yellow-throated martens feces could be classified into four groups: mammals, birds, insects, and plants [10]. However, their results were based on morphological identification, which has limited resolution at the species level, compared to molecular methods. Eurasian river otter is top of the aquatic food chains and is extremely sensitive to pollution and habitat destruction [11]. Eurasian river otter is not only a symbolic species and irreplaceable ecological function species of wetland and aquatic environment but also a health indicator of terrestrial freshwater systems (rivers, lakes, and mountain water systems) [11]. Eurasian river otter is a typical amphibious mammal, which has ascended to the top of the food chain and ecological function in wetland and aquatic environments of northeast China [12]. However, as apex predators in rivers, there are no data on its diet in China so far. Finally, Siberian weasels are widely distributed in China with mixed diets, feeding mainly on small mammals (e.g., voles, squirrels, mice, and pikas), amphibians, fish, and carrion, as well seasonally available pine *Pinus* nuts [13]; however, little is known of Siberian weasels in our study area.

Similar species are often found to partition resources where they are sympatric. An earlier study on yellow-throated marten and Siberian weasels indicated that they mainly prey on small mammals, with large mammals as secondary diet items [14]. Yellow-throated marten and Siberian weasel have a similar diet, niche breadth, and a high diet overlap, based on morphological species identification of stomach contents [14]. In general, the diets and food web relationships are still insufficiently characterized for the three sympatric Mustelidae species studied here. In addition, molecular diet analysis from feces may be easier to carry out as the three targeted species are not very common due to human disturbance and the destruction of their environment [15,16].

**Figure 1:**
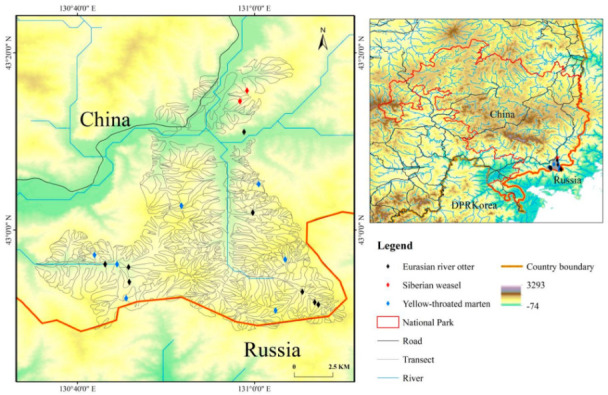
Location and habitat of the study area in the Northeast Tiger Leopard National Park in Northeast China. Different symbols indicate the localities of individual fecal samples from the mesopredators species used in the diet metabarcoding analysis.

Human activity is known to greatly impact local food webs and resource availability for mesopredators [15]. For example, cattle grazing reducing understory food resources directly impacts ungulate and rodent abundances in the east part of the Northeast Tiger and Leopard National Park of Northeastern China (Figure 1) [17]. Similarly, medical focused explorations have resulted in in amphibian reductions [18,19], while fish communities are directly impacted by overfishing [20], pesticide use, and dam building [21]. Importantly, as these prey communities are impacted, predator guilds are subsequently directly affected as well [17]. More directly, pesticide and herbicides use [22], construction of power stations, dams, and diversion channels all affect predator (e.g., Mustelidae species) activities and carrying capacities [23]. Consequently, more knowledge is needed to increase our understanding of impacts of environmental stressors on the diet composition of these species to develop refined conservation and management strategies. Recently developed next-generation, sequencing-based DNA metabarcoding has enabled highly sensitive, accurate, quantitative, and time-efficient dietary analysis of practically any food type at a fine taxonomic resolution, revealing essential information regarding interspecific competition and resource partitioning that could be missed when using morphological methods [5]. Moreover, molecular analysis of recovered feces overcomes the elusive nature of these Mustelidae species. Thus, we apply DNA metabarcoding to understand the diets of yellow-throated marten, Eurasian river otter, and Siberian weasel from the Northeast Tiger and Leopard National Park.

## 2. Materials and Methods

### 2.1. Study Area and Sample Collection

Our study was conducted in the northeastern part of Jilin Province in an area of 6501 km^2^ (130.165609° E–131.317653° E and 42.614041° N–43.553505° N) that is part of the Northeast Tiger Leopard National Park in northeast China (Figure 1). This area is on a rugged, mountainous landscape with elevation ranging from 5 to 1477 m above sea level (m a.s.l.) [24]. The main vegetation types include Korean pine (*Pinus koraiensis*) forests, oak forests, coniferous forests, natural shrublands, and agricultural areas. The region is home to apex predators that include Amur tiger (*Panthera tigris altaica*), Amur leopard (*Panthera pardus*), Asiatic black bear (*Ursus thibetanus*), brown bear (*Ursus arctos*), and Eurasian lynx (*Lynx lynx*). Additionally, mesopredators of the area include Asian badger (*Meles leucurus*), raccoon dog (*Nyctereutes procyonoides*), red fox (*Vulpes vulpes*), Siberian weasel, yellow-throated marten, Eurasian river otter, and leopard cat (*Prionailurus bengalensis*) [15]. In this park, yellow-throated marten lives in forest habitat, while Siberian weasel can be found in mountain forest habitat and agricultural and grasslands. Eurasian river otters currently occur in all types of aquatic ecosystems.

We designed 100 fixed line transects and collected scats matching the description of that of small carnivores along these transects from March 2021 to September 2021. These line transects were distributed from plain to deep mountains and forests in the core area of the national park. Each transect had a length of 10 km, and the distance between them was 0.1 km. Each region was about 10 square kilometers (km^2^); our study area contained 10 regions. So, the total area was 100 km^2^. The collected carnivore scats were submerged in 95% ethanol and stored at −20 °C until further processing. Genomic DNA from all the samples were isolated using the Qiagen Stool DNA extraction kit (Qiagen, Valencia, CA, USA), according to the manufacturer’s instruction manuals.

### 2.2. Molecular Species Identification

First, for the unambiguous identification of the three target Mustelidae species, a 350 bp fragment of the mitochondrial 16S rRNA gene was amplified by PCR using the primers 16S-F/16S-R [6] and subsequent comparison of resulting DNA sequences to DNA sequence data from reference taxa of GenBank. PCR was carried out in a total of 20 μL, using 2 µL of DNA extract as a template. The amplification mixture contained 2 × Taq PCR Super Mix (Innovagene Biotech, Changsha, China), 0.48 μ M16S-F/16S-R primers (Table 1), and 1 μg bovine serum albumin. The PCR mixture was denatured at 94 °C for 5 min, followed by 35 cycles of 30 s at 94 °C, 30 s at 53 °C, and 50 s at 72 °C, with a final elongation step of 2 min at 72 °C. The length of the amplification product was first revealed through implementing electrophoresis on a 2% agarose gel, and then PCR results were sent to Beijing Qingke Biotechnology Co., Ltd. (Beijing, China) for sequencing based on an ABI PRISM 3730 Genetic Analyzer [25]. The primers and ambiguous fragments in the sequencing products were removed by the Chromatogam software, and the obtained DNA sequence was compared with the reference sequences in BLAST (basic local alignment search tool) [26] database in Genbank [27]. When the sequence identity was higher than 98% [3], we considered the DNA sequence to belong to the respective species.

### 2.3. Diet Analysis

We conducted diet analyses for scat that was identified as coming from one of the three Mustelidae species (see above). Scat was hand-homogenized and DNA extracted using the Qiagen Stool DNA extraction kit, following manufacturers protocols. The 12SV5F/R (Table 1) primer pairs were used to amplify a fragment of ~100 bp of the V5 loop of vertebrate mitochondrial 12S rRNA gene [28], which has been demonstrated to have high species resolution power across most vertebrate taxa. To prevent the amplification of predator DNA, we designed a blocking oligonucleotide for each species (i.e., OSBI for Eurasian river otter, MartesB for yellow-throated marten, MustelaB for Siberian weasel) to efficiently amplify dietary species only, which was based on former study [29,30]. These oligonucleotides have a C3 spacer-CPG modification at the 3′-end that can specifically prevent the amplification of the originating carnivore’s DNA sequence [26].

The PCRs for generating dietary data were conducted in a total volume of 25 μL reactions with 4 μL extracted DNA (~10 ng/μL), 1 μm of 12SV5F/R, 4 mM blocking oligo, 0.4 mg/mL bovine serum albumin (BSA), and 2 × Taq PCR Super Mix (Innovagene Biotech, Changsha, China). The PCR program started the denaturation step with 5 min at 94 °C, followed by 35 cycles of 30 s at 94 °C and 30 s at 55 °C. As the target sequences were ~100 bp long, the elongation step was removed to reduce the +A artifact [31]. The 12SV5-F and 12SV5-R primers were tagged at the 5′-end with nine nucleotides beginning with CC followed by 7 variable nucleotides. These tags differed by at least three nucleotides among the tags, thus allowing a unique tag for each PCR to sort sequences according to samples following NGS. Seven PCR blanks (containing all PCR reagents except DNA) were also included in the amplifications to check for contamination. PCR products were purified using a PCR purification kit (EasyPure PCR Purification Kit, TransGen Biotech, Beijing, China). DNA concentrations were determined by agarose gel electrophoresis and using a NanoDrop 2000 spectrophotometer (ThermoScientific, Waltham, MA, USA) and were mixed in equimolar concentrations. PCR products were pooled in equal volumes for sequencing library construction. Next-generation sequencing (NGS) was carried out on the Illumina HiSeq X Ten platform (Illumina, San Diego, CA, USA) using paired-end sequencing, following the manufacturer’s instructions. A total of 100 nucleotides were sequenced on each end of the DNA fragment [5].

### 2.4. Data Analysis

The OBITools package [32] was used for sequence filtering and preliminary species identification. The forward and reverse sequences were aligned on the Illumina paired-end program, and aligned sequences with a quality score <40 were removed by the obigrep command. Sequences with perfectly matched tags and a maximum of two mismatches in primers were identified by the ngsfilter program and kept for further analysis. Sequences < 80 bp were removed using the obigrep program. PCR and sequencing errors were detected on the obiclean program [33]. The sequences of humans and the originating carnivores were removed, as well as low-frequency sequences (<0.1% or 50 reads in the sample or fewer than the count of the sequence in the extraction blanks and PCR blanks of the same library), which were potentially the results of contaminations and sequencing errors [34].

Automatic taxonomic assignments were checked by BLAST [27] using the NCBI nucleotide database and refined with species distribution information from the study areas based on the following criteria. For vertebrate sequences, only those with 100% coverage in the public databases were considered, using the following criteria for taxonomic assignment: (1) When the identity of a query was ≥99% with only one species’ sequence in the database, the query was assigned to this species; (2) When the identity of a query was ≥99% with more than one species’ sequence, we first checked its distribution; if more than one species was present in the study area, the lowest taxonomic level that included all the species was assigned; (3) When the query sequence identity matched between 92% and 99% with the DNA sequences in the database, the query was assigned to the lowest taxonomic level that could include all locally occurring species with the highest identity scores; if a single species showed the highest identity similarity but was not known to inhabit the area, the most closely related local species within the same genus was assigned; (4) When the maximum identity for a query was <92%, the taxon was recorded as unknown. To avoid misidentification of taxa due to insufficient local species records, we kept to conservative taxonomic assignments and only excluded species that showed no occurrence in all of northeast China [3].

The Mustelidae diet was quantified in two ways: (1) As a percent frequency of occurrence in fecal samples (%FC, if there are multiple food items in the scats) [6,16]; (2) As a proportion of occurrence of an individual food resource (%TX, range 0–1 for each food source) [6]. 

## 3. Results

**Figure 2:**
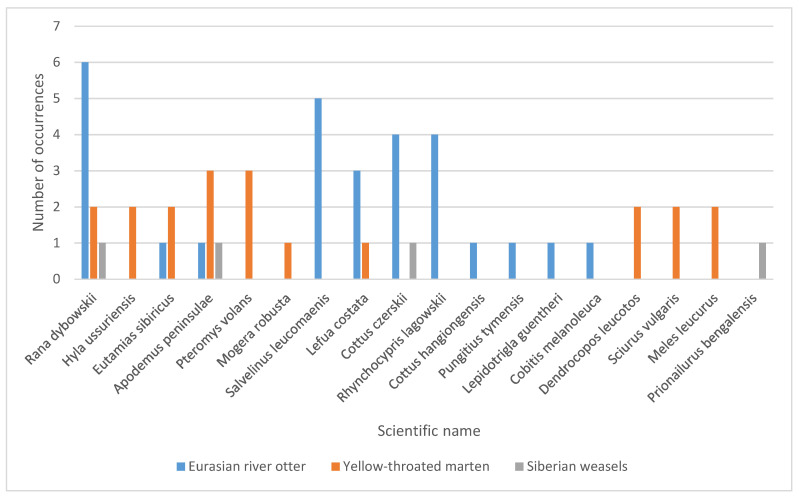
Vertebrate prey species molecularly identified in scats of three Mustelidae collected the Northeast Tiger Leopard National Park in northeast China.

A total of 527 scats were collected during a period of 4–8 months in 2021. Of these scats, we selected 350 carnivore scats according to their morphology and confirmed that they belonged to carnivores through a genetic species identification method (i.e., 16S; Table 1). Seven scats were confirmed to be that of the yellow-throated marten, eight were Eurasian river otter, two were Siberian weasel, while the other samples collected belonged to the red fox, leopard cat, Amur tiger, Amur leopard, Asiatic black bears, and Asian badger. After removing low-quality sequences, primer or tag sequences, sequencing errors, and unidentified reads, we collapsed the sequences into 317,947 unique sequences. Based on sequence similarity and the distribution of these target species’ prey in the study area, we identified 26 taxa to the species level, 2 to the genus level, and 1 to the order level among all scats (Figure 2).

**Figure 3:**
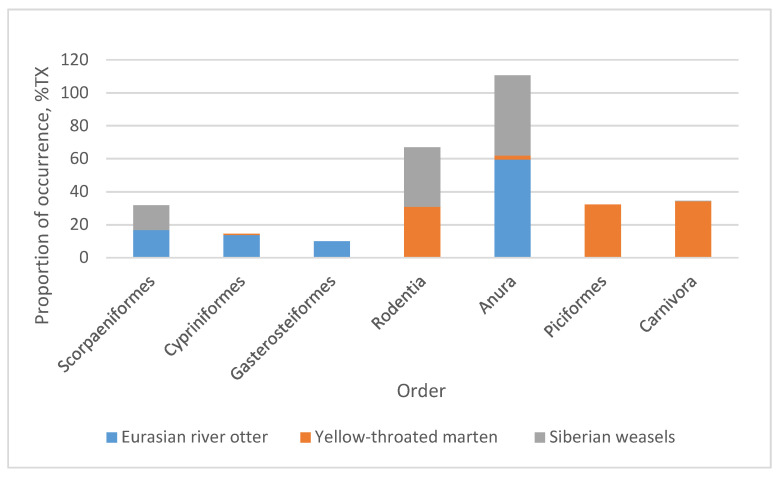
Proportion of occurrence of vertebrate prey orders in the diet of three Mustelidae collected in the Northeast Tiger Leopard National Park of northeast China.

Among the total 21 prey taxa, 10 were consumed by yellow-throated marten, 14 by Eurasian river otter, and 4 by Siberian weasel; some prey species were identified in more than 1 Mustelidae species. Prey included 7 mammalian taxa, 10 fishes, 2 birds, and 2 amphibians, with 85% (18/21) of the taxa assigned to the species level. Two vertebrate taxa prey were identified to the genus level; these were *Cottus* spp. and *Lampetra* spp. (in Eurasian river otter scats, %FC are 12.5% and 12.5%, respectively). One vertebrate prey item was identified to the Galliformes level (in Eurasian river otter scats, %FC = 12.5%). The maximum amount of prey items identified in a single scat sample of Eurasian river otter was six, in yellow-throated marten it was five, and in Siberian weasel it was two (Figure 2). Most frequently, Dybowski’s frog was found in 75% of Eurasian river otter fecal samples, followed by several fishes and rodents. Species in the order Anura were the primary food item of the Eurasian river otter when the samples were analyzed by the proportion of occurrence in individual prey taxa (%TX = 59.40%), followed by fish (40.79%) (Figure 3).

The most common prey taxa of yellow-throated marten were Siberian flying squirrel (*Pteromys volans*, %FC = 42.86%) and Korean field mouse (*Apodemus peninsulae*, 42.86%). The complete taxon list comprising the diet of the yellow-throated marten taxa is presented in Figure 2. Species in the orders carnivora (%TX = 34.2%), Piciformes (33.27%), and Rodentia (30.83%) were the most dominant prey of the yellow-throated marten (Figure 3). As for Siberian weasel, Dybowski’s frog was the primary food item (%TX = 48.76%), followed by Korean field mouse (35.94%) and Cherskii’s sculpin (14.94%) (Figure 2 and Figure 3). In addition, Galliformes (0.06% only in Eurasian river otter), Salmoniformes (0.17% only in Eurasian river otter), Petromyzonidae (0.03% only in Eurasian river otter), and Insectivora (0.36% only in yellow-throated marten) occurred in very low proportions and were not included in Figure 3.

Overall, there were two taxa (Dybowski’s frog and Korean field mouse) shared by all three Mustelidae species. The Dybowski’s frog proportion of occurrence in the diets of Eurasian river otter, yellow-throated marten, and Siberian weasel was 59.40%, 1.26%, and 48.75%, respectively.

## 4. Discussion

Here, we developed molecular methods that permit predator and prey determination from a single feces samples; a valuable practice where multiple apex and mesopredators exist. Specifically, we describe primer and PCR procedures that identify the predator prior to using blocking oligonucleotides to prevent the amplification of predator DNA when assessing prey from the same scat sample. Doing so offers detailed information on dietary items at the species level, and thus contributes to future investigations of food webs and niche partition in these Mustelidae and other predator species.

For Eurasian river otters, we recovered fish as the primary prey item, which is consistent with previous studies [35,36], though the specific fish species within the diet corresponds to those found in different geographical regions. In addition, we found frog species as a secondary prey item for Eurasian river otter. We note that our inability to recover any crustacean species as part of the Eurasian river otter’s diet, as was recovered in other studies [34], is likely due to the primers we used not amplifying the mitochondrial 12S rRNA gene in crustaceans. Future studies will require the design of a cocktail of primers that will be able to amplify across mammalian and non-mammalian prey items to get a complete understanding of diet. Moreover, future work will require the expansion of our study area and study periods to get more feces and reveal the food web relationships of all mesopredators in the Northeast Tiger Leopard National Park, including seasonal dietary shifts across time and space.

Scat site information indicated that the yellow-throated marten is distributed between an altitude of 262–523 m a.s.l., Eurasian river otter is found at an altitude of 185–392 m a.s.l., and Siberian weasel is distributed at an altitude of 332 m a.s.l. in our study area, which further suggests that they are sympatric species. There were differences in diet composition in different Mustelidae species, but some dietary items were also shared among species, which may indicate competition between these Mustelidae species. Among collected scats, there were other mesopredator species, in addition to the three targeted Mustelidae species. It is known that these three targeted species have lower population sizes than other predator species in the study area, as shown with infrared trigger camera data [37,38]. Together, we posit that the high carnivore biodiversity in our study area may explain population size differences as a result of competition from the spatiotemporal overlap and resulting complex food web relationships. Future studies can use the developed methods to focus on understanding these interspecific relationships that will help shed light on the role(s) of biotic interactions occurring within these mesopredator communities.

Direct and indirect human disturbances on the landscape have important and often negative consequences on mesopredator communities, including in our study area [7,17,39]. Thus, conservation initiatives can focus on recovering wild prey species through reducing or excluding livestock from the forest and minimizing the harvesting of wild frogs [39]. We suggest that efforts should also include the development of alternative livelihood opportunities for local communities that will eventually result in a reduced harvest of important prey species. Together, with provincial and national support, alternative economic and land use strategies can balance biodiversity, local development, and long-term goals to increase ecological services both at the local and national level [24]. In addition, we recommend establishing ecological corridors or expanding current reserve space is essential for the conservation of mesopredator communities.

## Figures and Tables

**Table 1:** Sequences of the primers used in this study. Product sizes do not include the primer sequences.

Name	Target Sequence	Product Size (bp)	Ta (°C)	Primer Sequences (5′-3′)	Reference
16S	Carnivore mitochondrial 16S rRNA gene	~350 bp	54	F-GAGAAGACCCTATGGAGC R-ATAGAAACCGACCTGGAT	Xiong et al., 2016 [6]
12SV5	Vertebrate mitochondrial 12S rRNA gene	~100 bp	55	F-TAGAACAGGCTCCTCTAG R-TTAGATACCCCACTATGC	Riaz et al., 2011 [28]
OSB1	otter mitochondrial 12S rRNA gene	~100 bp	56	CTATGCTCAGCCCTAAACATAGATAGCTTACATAACAAAACTATCTGCC-C3	This study
MartesB	yellow-throated marten mitochondrial 12S rRNA gene	~100 bp	55	CTATGCCCAGCCCTAAACACAAACAATTTACGTAACAAAATTGTCTG-C3	This study
MustelaB	Siberian weasel mitochondrial 12S rRNA gene	~100 bp	54	CTATGCTCAGCCCTAAACATAAATAATTATCACAACAAAATTATCTG-C3	This study

## Data Availability

The data presented in this study are available on request from the corresponding author.
